# Capsaicin, the Pungent Component of Red Chili Pepper, induces p21-mediated Cell Cycle Arrest in Renal Cell Carcinoma via Downregulating GLI1: an Experimental Research *In Vitro*

**DOI:** 10.7150/jca.119706

**Published:** 2025-10-20

**Authors:** Long Zheng, Yongchao He, Hao Huang, Wei Qu

**Affiliations:** 1 Department of Nuclear Medicine, the Second Affiliated Hospital of Xi'an Jiaotong University, Xi'an, Shaanxi, China.; 2 Xi'an Jiaotong University Health Science Center, Xi'an, Shaanxi, China.

**Keywords:** GLI1/p21, cell cycle arrest, capsaicin, proliferation, renal cell carcinoma

## Abstract

Capsaicin is the pungent and bioactive compounds of *Capsicum annum.* Previous studies have demonstrated the potent anti-tumor effect of capsaicin on various human malignancies by experiments *in vitro* or *in vivo*. However, the mechanism underlying its antitumor efficiency is not fully elucidated. Cell cycle arrest is one of anti-proliferation mechanisms for anti-neoplastic drugs. The p21 protein, an important inhibitor of cell cycle progression, could block cyclin dependent kinases (CDKs) mediated activation of cyclins. As the downstream transcriptional factor of the Hh pathway, GLI1 plays a crucial role in cancer progression and prognosis evaluation. The aim of the present study is to explore the potential role of GLI1 in p21 mediated cell cycle arrest induced by capsaicin treatment in renal cell carcinoma (RCC) 786-O and Caki-1 cell lines. The results revealed that capsaicin could inhibit proliferation of RCC cells and cause G_0_/G_1_ cell cycle arrest* in vitro*. Besides, we discovered that the capsaicin treatment increased the expression of p21 protein and downregulated the expression of GLI1, suggesting that GLI1 was involved in the p21 mediated G_0_/G_1_ arrest induced by capsaicin administration in RCC 786-O and Caki-1 cell lines. In conclusion, our study demonstrated that capsaicin could induce p21 mediated cell cycle arrest via suppressing GLI1 to inhibit RCC cell proliferation, which might be a promising therapeutic strategy in RCC.

## Introduction

Renal cell carcinoma (RCC) is heterogeneous cancer that may initiate from different tissues throughout the kidney 1. The latest global statistics of 36 common cancers show the incidence and mortality of RCC were respectively ranked 14^th^ and 16^th^, with 434,419 new cases and 155,702 deaths 2. Clear cell renal cell carcinoma (ccRCC) is accounted for 70-90% cases of RCC, and it is the most lethal subtype with a mortality rate of 30%-40% 3, 4. For early-stage RCC, surgical resection is the most crucial and effective treatment 5. However, since the metastatic RCC is resistant to radiotherapy and chemotherapy, there is no effective therapy to completely cure over the past three decades 6. Although immunotherapy and targeted therapy can retard tumor progression under a particular situation, the RCC patients will eventually suffer local recurrence, distant metastasis, and even death 7. Therefore, further researching novel and effective molecular drugs without side effects is of great significance in RCC prevention and treatment.

The capsicums are globally consumed as food flavorings and folk medicines for thousands of years. Comprehensive studies have proved that capsaicin, the pungent and bioactive compounds of red chili pepper (*capsicum annum*), exerts analgesic, antioxidant, anti-inflammatory, anti-obesity and immunomodulatory properties in multiple biochemical processes 8, 9. Besides, previous researches have demonstrated the potent anti-tumor effect of capsaicin on various human malignancies in *vitro* and *vivo* experiments 10-12. In a word, capsaicin has the great potential in development of drugs for human diseases.

The anti-tumor function of capsaicin has been demonstrated by previous literatures in restraining cancer cell proliferation, metastasis and angiogenesis of multiple cancer types 13. As to RCC, the ghost pepper (the content of capsaicin ranges from 2%~4%) was found to regulate multiple signaling molecules (Ras, p53, TGF-β, Wnt/β-catenin, calcium, etc.) and exhibited antiproliferation activity via inducing apoptosis through a complex interaction network 14. Besides, Que et al discovered capsaicin could trigger AMPK/mTOR-mediated autophagy, which attenuate the ability of distant metastasis of RCC 15. As well as apoptosis induction, cell cycle arrest is another anti-proliferation mechanism for anti-neoplastic drugs 16. However, the effect of capsaicin on cell cycle process in RCC and the regulatory mechanisms remains unknown and needs to be further researched.

The Hedgehog (Hh) pathway is deemed to play an essential role in initiation and progression of various cancers, including RCC 17-19. The glioma-associated oncogene 1 (GLI1) is identified as the key transcriptional factor of Hh pathway 20. When activated by SMO or other signaling, GLI1 can be translocated into nucleus and orchestrate expression of its downstream oncogenes, leading to cancer development and progression 21. GLI1 has been identified as a vital regulatory gene in cell cycle progression. A series of studies prove that GLI1 inhibition induced cell cycle arrest in different cancer types 22-24. In RCC, the regulatory effects of GLI1 on cell cycle progression are seldomly reported and the detailed mechanisms are incompletely elucidated.

Until now, the interactions among capsaicin, GLI1 and cell cycle regulation of RCC are not reported by previous literatures. In the present study, we performed a series of experimental and bioinformatic methods, aiming to preliminarily explore the underlying mechanisms.

## Materials and Methods

### Reagents and antibodies

Capsaicin (M2028) was purchased from Sigma-Aldrich (St. Louis, MO, USA) and was diluted in DMSO and stored as a 200 mmol/L stock solution. Anti-GLI1 rabbit polyclonal antibody and p21 rabbit polyclonal antibody were purchased from Proteintech (Chicago, IL, USA). Anti-glyceraldehyde-phosphate dehydrogenase (GAPDH) mouse monoclonal antibody was purchased from Santa Cruz Biotechnology (Santa Cruz, CA, USA). Anti-cyclin D1 mouse monoclonal antibody was obtained from Proteintech (Chicago, IL, USA). The dimethylsulfoxide (DMSO), propidium iodide (PI), RNase A and 4,6-diamidino-2-phenylindole (DAPI) were purchased from Sigma-Aldrich (St. Louis, MO, USA). Tris-base, NaCl, sodium dodecyl sulfate (SDS), nonided-P40, sodium deoxycholate, acrylamide-bisacrylamide, ammonium persulfate, tetramethyle-thylenediamine, Triton X-100, glycerol and paraformaldehyde were purchased from Amresco (Solon, OH, USA). Nitrocellulose membranes were purchased from Pall Corporation (Pansacola, FL, USA) and neutral absorbent paper was purchased from Bio-Rad Laboratories, Inc. (Hercules, CA, USA).

### Cell culture

Human renal clear cell carcinoma cell line 786-o and Caki-1 were obtained from the American Type Culture Collection (Manassas, VA, USA) and cultured in RPMI-1640 medium with 10% fetal bovine serum (FBS) (Gibco, Carlsbad, CA, USA) containing 1% of penicillin-streptomycin. The culture temperature is set at 37 °C, and the concentration of CO_2_ was kept at 5%.

### Cell proliferation assay

Cell viability was analyzed by MTT assay. Cells (8x10^3^/well) were seeded in 96-well culture plates and incubated overnight. Then, the cells were treated with capsaicin (0, 50, 100, 150 µM) for 2 days. After being washed once, 0.5 mg/ml of MTT was added and incubation was carried out at 37˚C. Four hours later, the culture medium was removed and DMSO was added to solubilize the formazan crystals. Finally, the absorbance was measured at a wavelength of 490 nm using a microplate auto-reader (Bio-Tek Instruments Inc., Winooski, VT, USA). Independent experiments were repeated in triplicate.

### Flow cytometric analysis

Flow cytometric analysis was applied to evaluate the cell cycle progression. After the indicated capsaicin treatments for 2 days, the cells in each group were harvested and washed twice and then 70% ethanol was used to fix the cells at -20˚C for at least 24 h. For cell cycle detection, the cells were washed twice and incubated with 50 µg/ ml propidium iodide (PI) and 50 µg/ml Rnase A at room temperature for 30 min. Then, flow cytometry was performed using a FACSCalibur system with CELLquest software version 3.3 (both from Becton Dickinson, San Jose, CA, USA), and the cell cycle distribution was calculated using ModFit LT software (version 3.0; Verity Software House, Topsham, ME, USA).

### Plasmid transfection

GLI1 cDNA was cloned into the pcDNA3.1 vector. Cells achieved 70-80% confluence for plasmid transfection and were transfected with XtremeGene HP DNA or transfection reagents (Roche Diagnostics, GmbH) for 2-3 days, and harvested for subsequent experiments.

### Western blot

The capsaicin treated cell lines (786-o and Caki-1) were harvested and washed by cold PBS three times. Total cellular protein lysates were treated with radioimmunoprecipitation assay buffer [50 mM Tris (pH 8.0), 150 mM NaCl, 0.1% SDS, 1% nonided-P40 and 0.5% sodium deoxycholate] including proteinase inhibitors (1% cocktail and 1 mM phenylmethanesulfonyl fluoride). Then, the lysates were centrifuged at 20,000g for 15 min at 4˚C and the supernatants were collected. The protein concentration was quantified using an Enhanced BCA Protein Assay kit (Beyotime Institute of Biotechnology, Haimen, China). The subsequent western blot analysis was performed according to the experiment steps applied by our previous work 25, 26. The GAPDH was used as the loading control.

### Immunohistochemical analysis and survival analysis

Validation of protein expression of candidate genes was conducted in the Human Protein Atlas (HPA) database, which is an open accessed website providing protein or RNA expression data of normal and pathology tissues, cells, blood samples (https://www.proteinatlas.org/). The survival analysis was performed by the Kaplan-Meier Plotter (KM Plotter) which is built and maintained for assessing the survival effects of over 54,000 genes (mRNA, miRNA, protein) in 21 cancer types 27. The RCC dataset including 502 samples was chosen to explore the influence of GLI1 expression level on overall survival (OS) and disease-free survival (DFS). The hazard ratio (HR), log-rank P-value, and survival plots were computed and drawn by the website automatically.

### Statistical analysis

Data are expressed as the mean ± SE from three independent experiments. Statistical analysis was carried out by SPSS software (SPSS Inc., Chicago, IL, USA). The one-way ANOVA test was applied to showing the differences among groups. P < 0.05 was considered to indicate a statistically significant result.

## Results

### Capsaicin inhibits the proliferation of ccRCC cell lines

**Figure 1:**
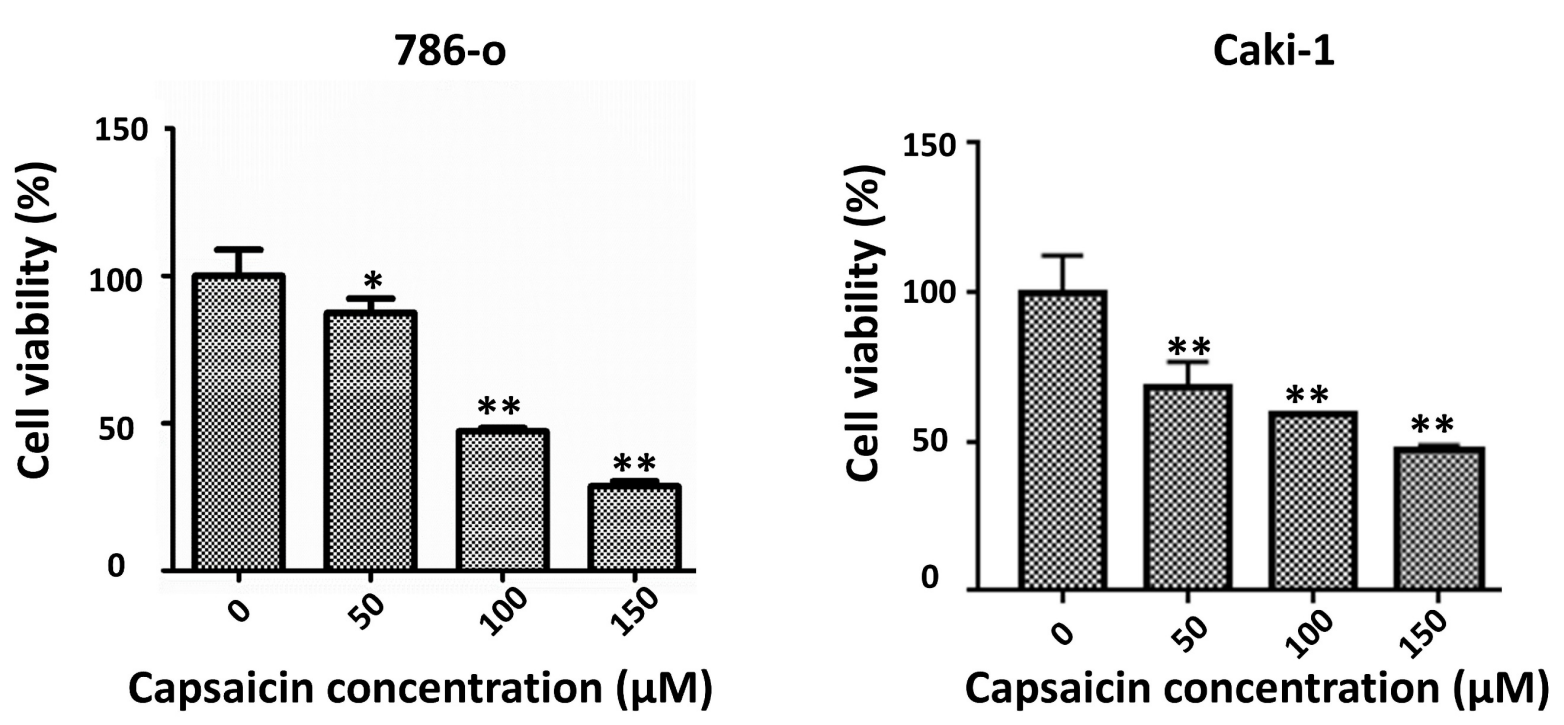
** Capsaicin inhibits ccRCC cell proliferation.** (A and B) MTT assay was used to detect the cell viability of 786-o and Caki-1 cells following treatment by capsaicin. The bar graphs showing cell viability were constructed by GraphPad Prism software. ccRCC, clear cell renal cell carcinoma; *P<0.05; **P<0.01.

In order to verify whether capsaicin affects the proliferation of ccRCC cells, we analyzed the effects of capsaicin on two RCC cell lines: 786-o and Caki-1. The cell lines were grown in RPMI-1640 medium with 10% FBS and treated with capsaicin at different concentrations for 48 h. Cell viability was then examined by MTT assay. Capsaicin induced a dose-dependent inhibition of cell proliferation in both RCC cell lines (Figure 1). The MTT assay showed that capsaicin inhibited cell proliferation in a dose-dependent manner. These results demonstrate that capsaicin inhibits the proliferation of ccRCC cells.

### Capsaicin induces G0/G1 cell cycle arrest in ccRCC cells

**Figure 2:**
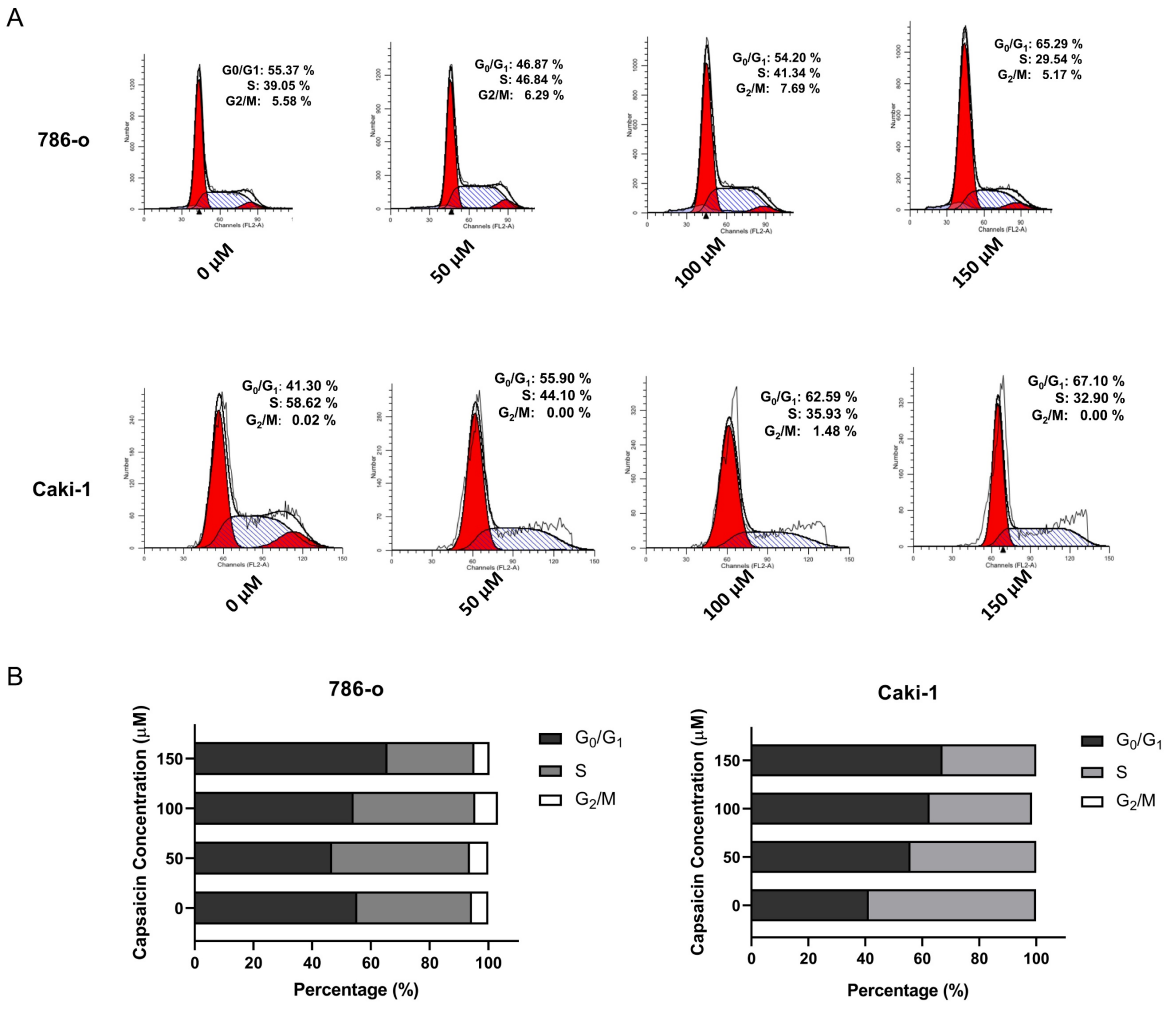
**Capsaicin inhibits ccRCC cell proliferation by inducing G0/G1 cell cycle arrest.** (A and B) The cell cycle distribution of the 786-o and Caki-1 cells were assessed by flow cytometry. (C) The bar graph showing the percentage of each phase of the cell cycle was constructed by GraphPad Prism software.

**Figure 3:**
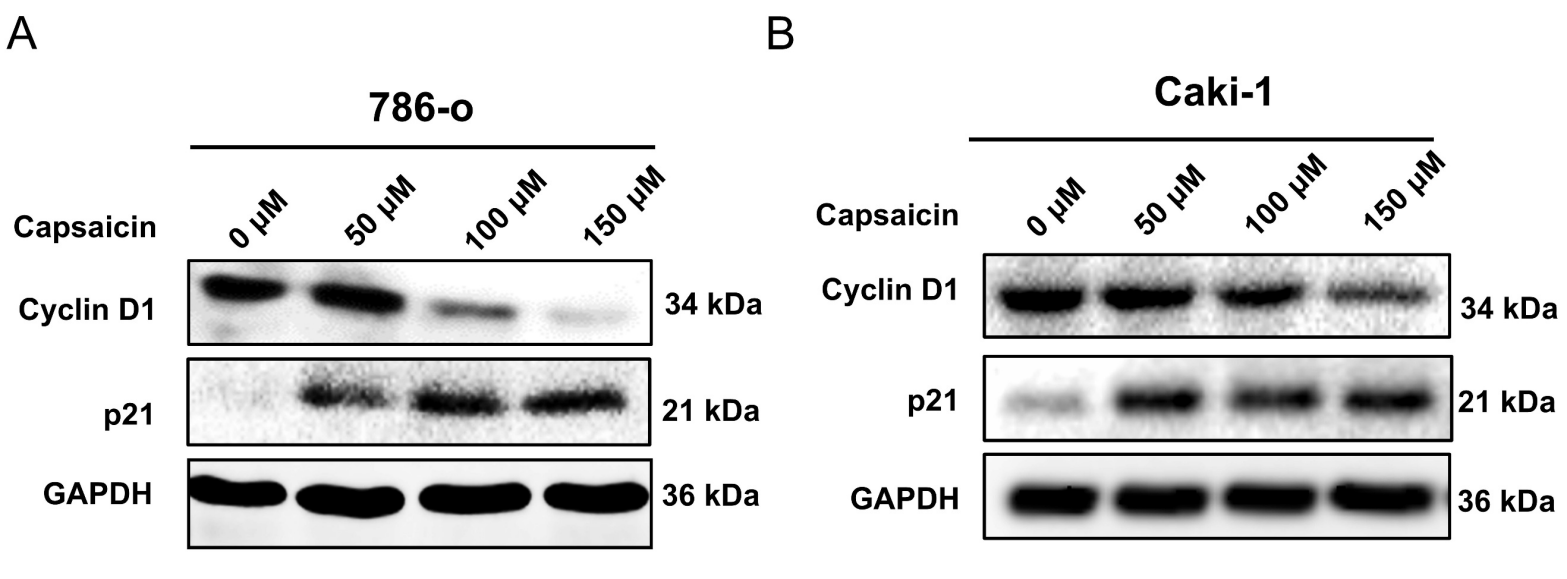
**Capsaicin induces expression profile changes of cell cycle regulating proteins.** (A and B) The level of cell cycle regulating proteins, cyclin D1 and p21, were detected by western blot analysis.

We further investigated the effects of capsaicin on cell cycle progression by flow cytometry. The 786-o and Caki-1 cells, in a logarithmic growth phase, were treated with concentration gradient of capsaicin (0,50,100,150μM) for 48 h. A significant increase in the percentage of cells arrested in the G0/G1 phases in both cell lines was observed; In parallel, there was a reduction in the percentage of cells in the S and G2/M phases (Figure 2). To confirm these data, western blot analysis was used to examine the expression of cyclin D1. Following treatment with capsaicin, the cyclin D1 was obviously reduced both in the 786-o and Caki-1 cells (Figure 3).

The p21 protein plays a crucial role in cell cycle regulation, mainly by inhibiting cell cycle-dependent kinases (CDKs) and downregulating the expression level of cyclins (such as cyclin D1) to inhibit the progression of the cell cycle. In the present study, our western blot analysis show capsaicin upregulated the p21 protein in a dose-independent manner (Figure 3).

Collectively, these results demonstrated that capsaicin affected the expression of key proteins involved in the cell cycle and induced G0/G1 cell cycle arrest in the RCC cells.

### GLI1 is identified as an upregulated gene and correlated with worse clinical outcomes in ccRCC patients by bioinformatic analysis

**Figure 4:**
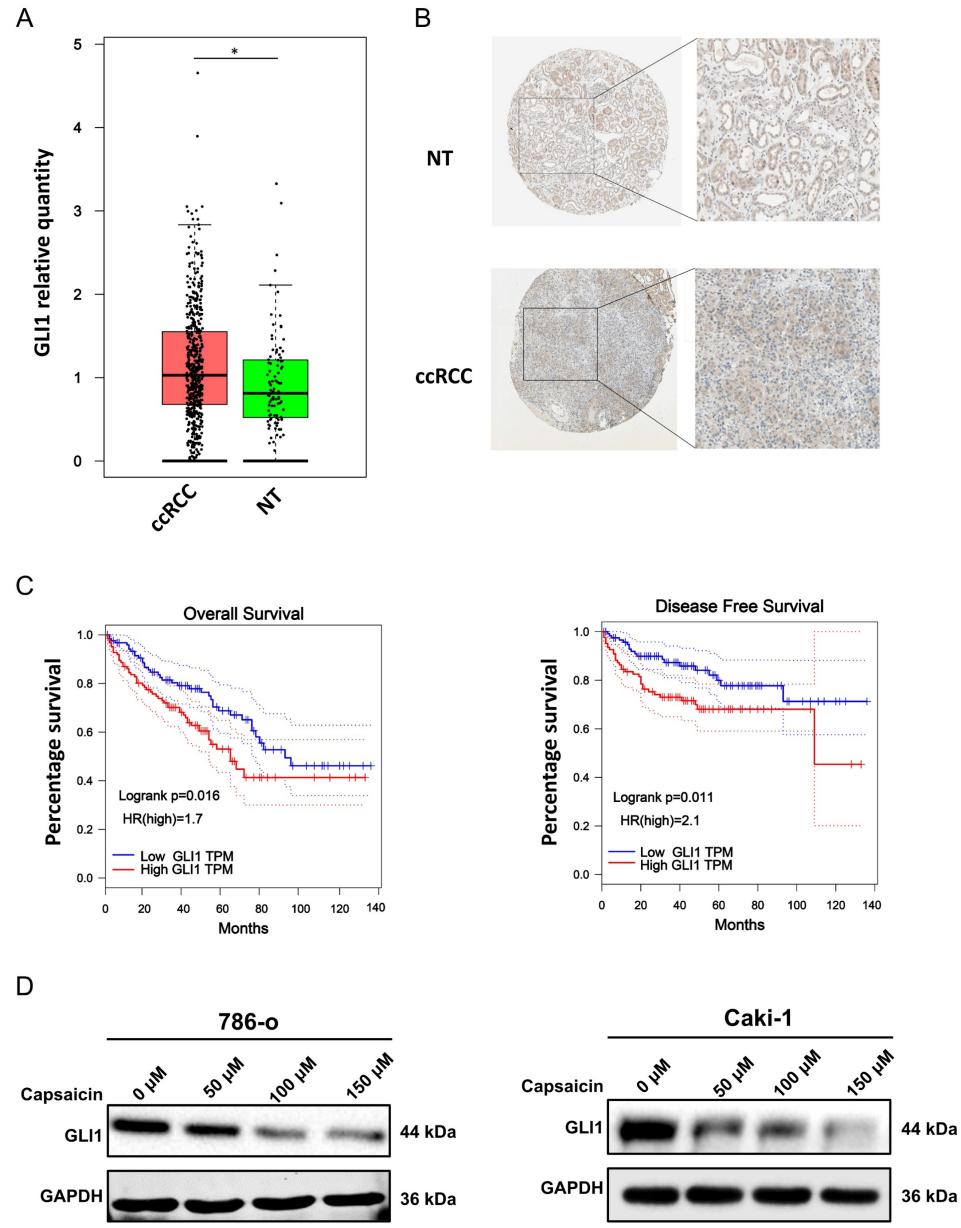
** Validating the expression profile and survival prediction of Gli-1 in ccRCC by bioinformatic analysis, and the influence of capsaicin on Gli-1 protein expression.** (A) Gli-1 mRNA expression profile in ccRCC based on data from the GEPIA database; (B) Gli-1 protein expression profile in ccRCC; (C) High Gli-1 expression is associated with short OS and DFS in ccRCC patients, according to the data from the KM-plotter database; (D) The influence of capsaicin treatment on Gli-1 protein expression profile was detected by western blot analysis. ccRCC, clear cell renal cell carcinoma; NT, normal tissue.

Intending to explore the expression profile of GLI1 in RCC, we performed differentially expressed gene analysis in GEPIA and HPA database. As shown in Figure 4A, GLI1 mRNA was highly expressed in RCC tissues compared with adjacent normal tissues. According to the results from the HPA database, GLI1 protein exhibits a significantly higher expression profile in ccRCC tissues than in normal tissues (NT) (Figure 4B). To explore the associations between GLI1 expression and ccRCC clinical outcome, we conducted survival analysis in the KM-plotter database. As shown in Figure 4C, abundant GLI1 expression correlated with OS and DFS in RCC patients compared with low expression patients.

### Capsaicin decreases the GLI1 protein expression in a dose-dependent manner

Aiming to explore the influence of capsaicin treatment on GLI1 expression, we performed western blot analysis and the results demonstrated that expression of GLI1 was decreased in a dose-dependent manner following treatment with the indicated capsaicin concentrations in these two cell lines (Figure 4D).

### Capsaicin enhances p21-mediated cell cycle arrest via downregulating GLI1

**Figure 5:**
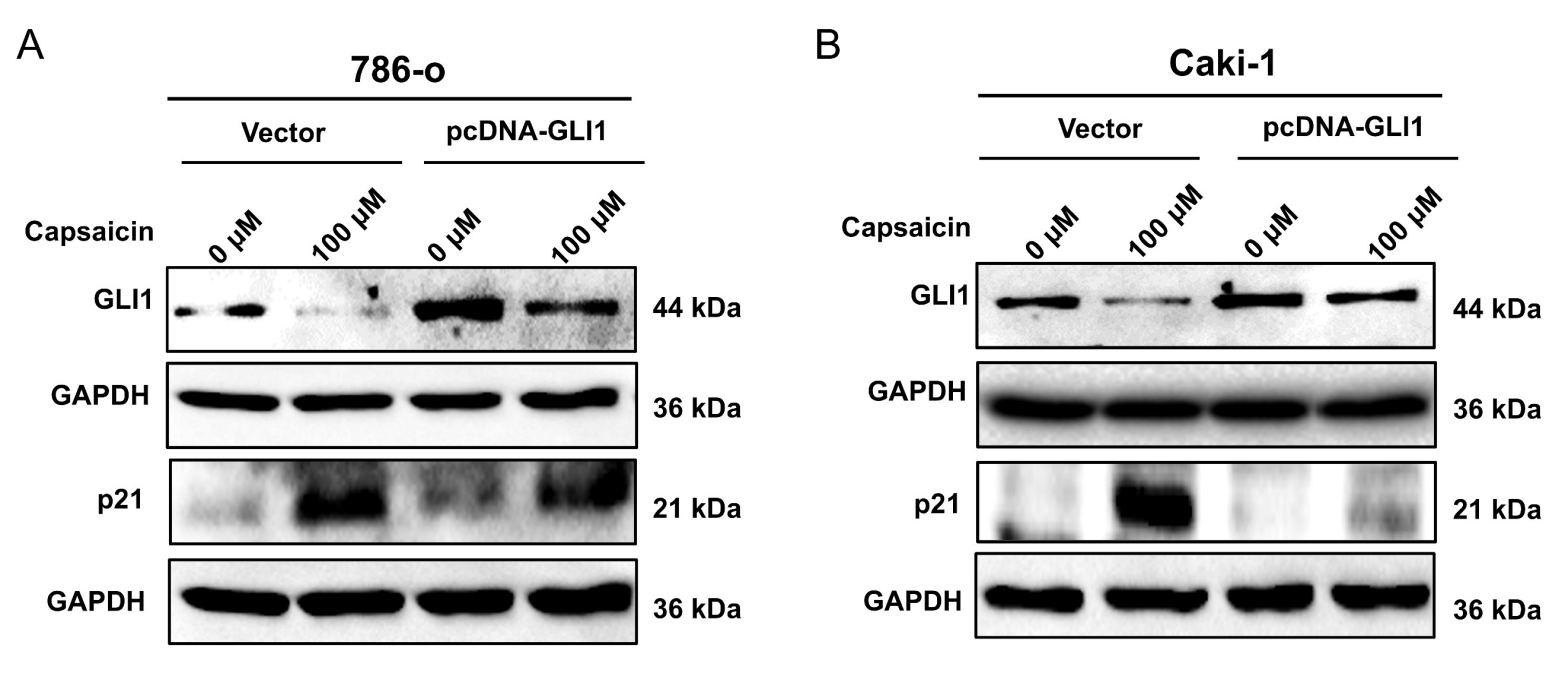
** Capsaicin induces overexpression of p21 through downregulating Gli-1.** The expression levels of Gli-1 and p21 were detected by western blot analysis in 786-o (A) and Caki-1 (B) ccRCC cell lines.

We overexpressed GLI1 by pcDNA3.0 plasmid in 786-o and Caki-1 cells and treated them with capsaicin (100 μM). The western blot result showed that the level of p21was upregulated, as compared with the level in the control group in both cancer cell lines (Figure 5).

## Discussion

It is widely acknowledged that bioactive components extracted from plants are potential therapeutic medicines for malignancies due to their relatively nontoxic and safe profiles 28. Capsaicin is a typical member of them and has been globally applied in diet for thousands of years. Therefore, capsaicin has been researched in various tumors because of it antitumor effects. RCC is the relatively intractable cancer among urinary tumors since it is resistant to conventional therapies in clinics, and the efficacy of the novel immune-targeted therapy for RCC is also not satisfactory 29. It thus appears that completely exploring the pharmacology of capsaicin on RCC is of great academic significance to early realization of capsaicin as a novel anti-RCC drug.

In the present study, we performed MTT assay and found the administration of capsaicin with a concentration gradient significantly decreased the numbers of viable RCC cell* in vitro*. Since our previous studies indicated capsaicin had no apparent effect on apoptosis 26, we determined to apply cell cycle analysis. The results show capsaicin induced G0/G1 arrest in RCC cell lines and the cycle -related proteins, such as cyclin D1, was downregulated. The p21 is identified as an inhibitor of cyclin dependent kinases (CDKs). It could block CDKs mediated progression of cyclins after being upregulated and activated 30. The outcome of western blot discovered that p21 was upregulated by capsaicin treatment, suggesting capsaicin restrain RCC proliferation by activating p21-mediated cell cycle arrest.

As the downstream transcriptional factor of the Hh pathway, GLI1 plays a crucial role in cancer progression and prognosis evaluation, while it is activated by an SMO-dependent or independent manner. The outcome of bioinformatic analysis performed by us validated that GLI1 was more highly expressed in RCC tissues than normal tissue, whether at the mRNA level or the protein level. We also found that RCC patients with highly expressed GLI1 exhibited shorter overall survival and disease-free survival, compared to those with lowly expressed GLI1. Relevant researches reported that high GLI1 level also predicted poor prognosis in malignant mesothelioma 31, gastric cancer 32, hepatocellular carcinoma 33, and other cancer types. In addition, GLI is an important enabler of cell cycle. According to the literatures that respectively launched by Wang K 34, Sun Y 35 and Sun J 36, GLI1 inhibition arrested cell cycle at G_0_/G_1_ or G_2_/M phase in cancers of brain, cartilage, and esophagus. In the present study, capsaicin treatment significantly downregulated expression level of GLI1 protein, indicating that Gli-1 maybe involve in capsaicin-induced cell cycle arrest in RCC.

**Figure 6:**
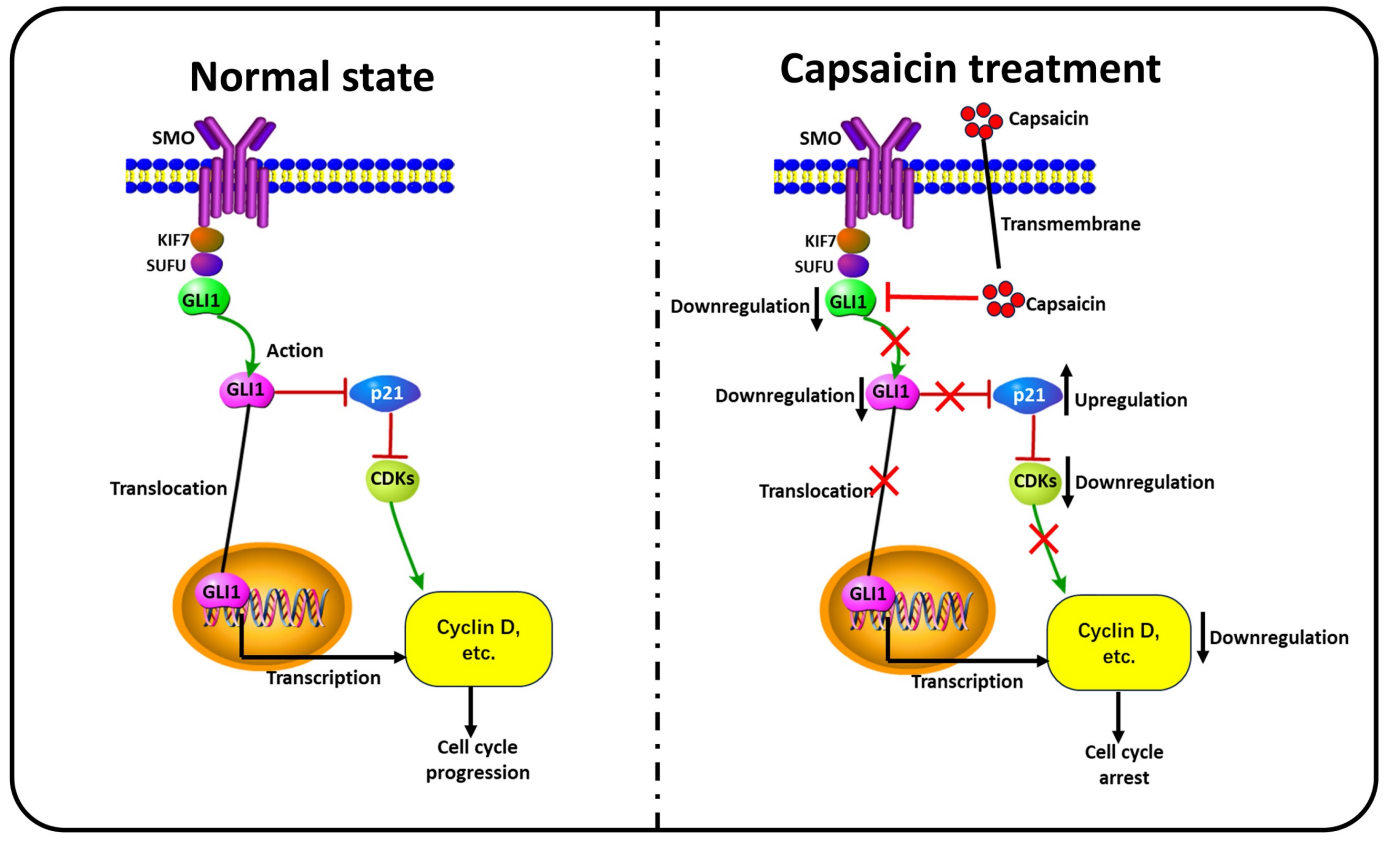
**Summary of the mechanisms involved in the capsaicin-mediated cell cycle arrest on ccRCC cells.** Under the normal condition, Gli-1 is activated by Hh signal and translocates into the nucleus. The activated Gli-1 binds to the DNA promoter region and upregulates the expression of cyclin D1. Meanwhile, the activated Gli-1 could suppress the expression of the p21 protein, relieve the inhibitory status of downstream cyclin-dependent kinases (CDKs), and further activate cyclins, thereby maintaining cell cycle progression. In the capsaicin treatment condition, the drug can directly inhibit expression and activation of Gli-1; Then, prevent the nuclear translocation capacity and transcript promoting function of Gli-1. Simultaneously, capsaicin can also reverse Gli-1mediated p21 downregulation, and subsequently inhibit the activity of downstream CDKs, and then reduce the expression and activity of cyclins, thereby inducing cell cycle arrest.

As mentioned above, GLI1 and p21 were involved in RCC cell cycle arrest induced by capsaicin. Our experiment result show overexpression of GLI1 by pcDNA 3.0 plasmid restrained the capsaicin-induced elevation of p21 protein. Thus, we indicated capsaicin could induce p21 related cell cycle arrest by suppressing GLI1. The relevant mechanism diagram was exhibited in Figure 6. However, the underlying mechanisms of GLI1/p21 signaling the was not completely elucidated and needs further study in our future work.

## Conclusion

The present study confirmed that capsaicin could induce p21 mediated cell cycle arrest via suppressing GLI1 to inhibit RCC cell proliferation. GLI1 is a potential target for capsaicin in RCC, which has not reported by previous studies. Our findings may provide preliminary experimental evidence for clinical application of capsaicin in RCC patients.

## Abbreviations

RCC: renal cell carcinoma
ccRCC: clear cell renal cell carcinoma
Hh: Hedgehog
GLI1: glioma-associated oncogene 1
CDK: cyclin dependent kinase
MTT: 3-(4,5)-dimethylthiahiazo (-z-y1)-3,5-diphenyltetrazoliumromide
HPA: Human protein atlas
OS: Overall survival
DFS: disease-free survival
HR: hazard ratio

## References

[1] Bahadoram S, Davoodi M, Hassanzadeh S (2022). Renal cell carcinoma: an overview of the epidemiology, diagnosis, and treatment. G Ital Nefrol.

[2] Sung H, Ferlay J, Siegel RL (2021). Global Cancer Statistics 2020: GLOBOCAN Estimates of Incidence and Mortality Worldwide for 36 Cancers in 185 Countries. CA Cancer J Clin.

[3] Warren AY, Harrison D (2018). WHO/ISUP classification, grading and pathological staging of renal cell carcinoma: standards and controversies. World J Urol.

[4] Capitanio U, Bensalah K, Bex A (2019). Epidemiology of Renal Cell Carcinoma. Eur Urol.

[5] Hora M, Eret V, Trávníček I (2016). Surgical treatment of kidney tumors - contemporary trends in clinical practice. Cent European J Urol.

[6] Hsieh JJ, Purdue MP, Signoretti S (2017). Renal cell carcinoma. Nat Rev Dis Primers.

[7] Gul A, Rini BI (2019). Adjuvant therapy in renal cell carcinoma. Cancer.

[8] Zheng J, Zhou Y, Li Y (2016). Spices for Prevention and Treatment of Cancers. Nutrients.

[9] Nilius B, Appendino G (2013). Spices: the savory and beneficial science of pungency. Rev Physiol Biochem Pharmacol.

[10] Sarkar A, Das S, Rahaman A (2020). Eugenol and capsaicin exhibit anti-metastatic activity via modulating TGF-β signaling in gastric carcinoma. Food Funct.

[11] Thoennissen NH, O'Kelly J, Lu D (2010). Capsaicin causes cell-cycle arrest and apoptosis in ER-positive and -negative breast cancer cells by modulating the EGFR/HER-2 pathway. Oncogene.

[12] Dou D, Ahmad A, Yang H (2011). Tumor cell growth inhibition is correlated with levels of capsaicin present in hot peppers. Nutr Cancer.

[13] Clark R, Lee SH (2016). Anticancer Properties of Capsaicin Against Human Cancer. Anticancer Res.

[14] Perla V, Nadimi M, Reddy R (2018). Effect of ghost pepper on cell proliferation, apoptosis, senescence and global proteomic profile in human renal adenocarcinoma cells. PLoS One.

[15] Que T, Ren B, Fan Y (2022). Capsaicin inhibits the migration, invasion and EMT of renal cancer cells by inducing AMPK/mTOR-mediated autophagy. Chem Biol Interact.

[16] Wu H, Chen L, Zhu F (2019). The Cytotoxicity Effect of Resveratrol: Cell Cycle Arrest and Induced Apoptosis of Breast Cancer 4T1 Cells. Toxins (Basel).

[17] Kumar A, Kumari N, Gupta V (2018). Renal Cell Carcinoma: Molecular Aspects. Indian J Clin Biochem.

[18] Skoda AM, Simovic D, Karin V (2018). The role of the Hedgehog signaling pathway in cancer: A comprehensive review. Bosn J Basic Med Sci.

[19] Tang C, Li L, Xu Q (2023). ACKR3 orchestrates Hedgehog signaling to promote renal cell carcinoma progression. Mol Carcinog.

[20] Ingham PW (2022). Hedgehog signaling. Curr Top Dev Biol.

[21] Carballo GB, Honorato JR, de Lopes GPF (2018). A highlight on Sonic hedgehog pathway. Cell Commun Signal.

[22] Ahmad A, Tiwari RK, Saeed M (2022). Carvacrol instigates intrinsic and extrinsic apoptosis with abrogation of cell cycle progression in cervical cancer cells: Inhibition of Hedgehog/GLI signaling cascade. Front Chem.

[23] Khan F, Pandey P, Ahmad V (2020). Moringa oleifera methanolic leaves extract induces apoptosis and G0/G1 cell cycle arrest via downregulation of Hedgehog Signaling Pathway in human prostate PC-3 cancer cells. J Food Biochem.

[24] Wang S, Ran L, Zhang W (2019). FOXS1 is regulated by GLI1 and miR-125a-5p and promotes cell proliferation and EMT in gastric cancer. Sci Rep.

[25] Zheng L, Chen J, Ma Z (2016). Capsaicin enhances anti-proliferation efficacy of pirarubicin via activating TRPV1 and inhibiting PCNA nuclear translocation in 5637 cells. Mol Med Rep.

[26] Zheng L, Chen J, Ma Z (2015). Capsaicin causes inactivation and degradation of the androgen receptor by inducing the restoration of miR-449a in prostate cancer. Oncol Rep.

[27] Nagy Á, Lánczky A, Menyhárt O (2018). Validation of miRNA prognostic power in hepatocellular carcinoma using expression data of independent datasets. Sci Rep.

[28] Goyal S, Gupta N, Chatterjee S (2017). Natural Plant Extracts as Potential Therapeutic Agents for the Treatment of Cancer. Curr Top Med Chem.

[29] Chen YW, Wang L, Panian J (2023). Treatment Landscape of Renal Cell Carcinoma. Curr Treat Options Oncol.

[30] Engeland K (2022). Cell cycle regulation: p53-p21-RB signaling. Cell Death Differ.

[31] Ma GY, Shi S, Sang YZ (2023). High Expression of SMO and GLI1 Genes with Poor Prognosis in Malignant Mesothelioma. Biomed Res Int.

[32] Shao X, Cheng Z, Xu M (2019). Prognosis, Significance and Positive Correlation of Rab1A and p-S6K/Gli1 Expression in Gastric Cancer. Anticancer Agents Med Chem.

[33] Zhang J, Tu K, Yang W (2014). Evaluation of Jagged2 and Gli1 expression and their correlation with prognosis in human hepatocellular carcinoma. Mol Med Rep.

[34] Wang K, Pan L, Che X (2010). Gli1 inhibition induces cell-cycle arrest and enhanced apoptosis in brain glioma cell lines. J Neurooncol.

[35] Sun Y, Guo W, Ren T (2014). Gli1 inhibition suppressed cell growth and cell cycle progression and induced apoptosis as well as autophagy depending on ERK1/2 activity in human chondrosarcoma cells. Cell Death Dis.

[36] Sun J, Wang D, Li X (2017). Targeting of miR-150 on Gli1 gene to inhibit proliferation and cell cycle of esophageal carcinoma EC9706. Cancer Biomark.

